# Biological activities, isolated compounds and HPLC profile of *Verbascum nubicum*

**DOI:** 10.1080/13880209.2019.1643378

**Published:** 2019-08-10

**Authors:** Heba Abd El Hady El Gizawy, Mohammed Abdalla Hussein, Essam Abdel-Sattar

**Affiliations:** a Pharmacognosy Department, Faculty of Pharmacy, October 6 University, Sixth of October City, Egypt;; b Biochemistry Department, Faculty of Applied Medical Sciences, October 6 University, Sixth of October City, Egypt;; c Pharmacognosy Department, Faculty of Pharmacy, Cairo University, Giza, Egypt

**Keywords:** Liver biomarkers, inflammatory mediators, antiulcer activity, peritoneal macrophage cells

## Abstract

**Context:** Genus *Verbascum* (Scrophulariaceae) comprises about 360 species of flowering plants. *Verbascum* has been used in traditional medicine as an astringent, antitussive, analgesic and anti-inflammatory.

**Objective:** Nothing was found in the available literature concerning *Verbascum nubicum* Murb; therefore, the study evaluates the biological activities, isolated compounds and HPLC profile.

**Materials and methods:** Methanol extract (VME) and butanol fraction (VBF) of air-dried powdered *V. nubicum* were obtained. Four compounds were isolated from VBE and identified by ^1^H- and ^13^C-NMR. High-performance liquid chromatography (HPLC) profile was determined for (VME). LD_50_, *in vitro* antioxidant, *in vivo* antiulcerogenic and anti-inflammatory activities as well as hepatoprotective activity were assessed. Anti-ulcerogenic and hepatoprotective activities were supported by histopathological examinations.

**Results:** HPLC analysis of VME revealed the presence of luteolin 7-glucoside (2215.43 mg/100 g), hesperidin (954.51 mg/100 g) and apigenin (233.15 mg/100 g) as major compounds. Four compounds were isolated and confirmed by NMR data, were identified as gentiopicroside, luteolin, aucubin and gallic acid. The LD_50_ of VME and VBF extracts were calculated to be 8200 and 4225 mg/kg b.w., respectively. IC_50_ values of VBE and VMF as measured by DPPH·method were 43.6 and 50 µg/mL, respectively. Also, anti-inflammatory effect of VME (400 mg/kg b.w.) and VBF (200 mg/kg b.w.) induced edema model after 120 min were 61.93 and 56.13%, respectively. Antiulcerogenic activity of VME (400 mg/kg b.w.) and VBF (200 mg/kg b.w.) in albino rats were 65.14 and 84.57%, respectively.

**Conclusions:** The *V. nubicum* extracts displayed safe and promising antioxidant, anti-inflammatory and hepatoprotective properties. It can be also applied in the pharmacy industry, food industry and healthcare.

## Introduction

The genus *Verbascum* is one of the largest genera of the Scrophulariaceae family, comprising about 360 species of flowering plants. Traditionally *Verbascum* spp. was used to treat inflammatory disease, asthma, spasmodic cough and migraine (Zhao et al. 2011). It also has been used in folk medicine for coughs as infusions (Dimitrova et al. 2013). *Verbascum* species contain different classes of biologically active compounds such as flavonoids, phenyl ethanoids, saponins, sterols and iridoid glycosides (Akdemir et al. 2003). *Verbascum nubicum* Murb. is a biennial herbaceous species indigenous to Sinai region of Egypt and Al-Taif governorate of Saudi Arabia. Nothing was reported in the available literature concerning this species; either the active constituents or the biological activities; therefore, it seems worthwhile to explore this plant.

As an extension of our studies on medicinal chemistry research of some new biologically active natural products (El-gizawy and Hussein 2017; Hussein et al. 2017), we now report the biological activities, isolated compounds and HPLC profile of *V. nubicum* which might contain compounds with promising antioxidant, anti-inflammatory and antiulcerogenic activities.

## Materials and methods

### Instruments and materials

The NMR spectral data were measured using JEOL GX-600 (500 and 100 MHz) and Bruker (400 and 100 MHz) for ^1^H and ^13^C-NMR, respectively.

Indomethacin, formalin, acetic acid and LPS were obtained from Merck Ltd., Germany. DPPH and ABTS^•+^ were obtained from Sigma Chemical Company, USA. All other diagnostic kits, chemicals and solvent are of high grade.

#### Plant material

The aerial parts of *V. nubicum* were collected from Al-Taif governorate, Saudi Arabia, during February 2015 and identified by Prof. Abdulrahman S. Hajar, Department of Biology, Faculty of Science, King Abdulaziz University, Saudi Arabia. Voucher specimens (No. 1/1/2014) were deposited in the herbarium of Faculty of Pharmacy, King Abdulaziz University.

#### Preparation of extract

The dried aerial parts of *V. nubicum* were powdered (750 g) and extracted with MeOH using Ultra turrax homogenizer (3 × 2000 mL). The solvent was distilled off under reduced pressure and the dried methanol extract was kept at 4 °C (65 g). The crude MeOH extract (35 g) was suspended in water and partitioned with CHCl_3_ (3 × 200 mL) followed by *n*-butanol saturated with water (3 × 200 mL) to give 10.8 and 23.5 g, respectively. The *n*-BuOH fraction (23.5 g) was fractionated over Diaion HP-20 eluting with H_2_O and gradient MeOH-H_2_O mixtures (25–100%) to afford six main fractions (Fr 1–6); fractions were combined according to their TLC patterns. Fractions Fr-2 (60 mg), Fr-4 (80 mg) and Fr-6 (50 mg) were subjected to repeated chromatography on Sephadex LH-20 columns, using CHCl_3_–MeOH mixtures to afford pure compounds **1**–**4**.

***Compound 1*** was obtained as a white powder (4 mg).

^1^H-NMR (MeOD, 400 MHz): *δ*_H_ 5.78 (1H, m, H-8), 7.44 (1H,d, *J* = 1.6 Hz, H-3) 3.15(1H, dd, *J* = 9.2,8 Hz, H-9), 4.67(1H, d, *J* = 8 Hz, H-1′), 5.02 (1H, m, H-7a), 5.10 (1H, m, H-7b), 5.21–5.28 (2H, m, H-10), 5.64 (1H,m, H-6), 5.68 (1H, d, *J* = 2.8 Hz, H-1). ^13^C-NMR (MeOD, 100 MHz): *δ*_C_ 98.9(C-1), 150 (C-3), 103.5(C-4), 127(C-5), 118(C-6),70(C-7), 135(C-8), 46.5 (C-9), 117(C-10), 166 (C-11), 100 (C-1′), 74.6(C-2′), 78(C-3), 72 (C-4′), 79(C-5′), 63.5(C-6′).

***Compound 2*** was obtained as a yellow powder (10 mg).

^1^H-NMR (DMSO, 400 MHz): *δ*_H_ 12.95 (1H, s, 5-OH), 7,42 (d, 2H, H-2′,6′), 6.90 (d, *J* = 8.0 Hz, 1H,H-5′), 6.69 (1H, s, H-3), 6.44 (1H, d, *J* = 2.0 Hz, H-8), 6.19 (1 H, d, *J* = 2.0 Hz, H-6). ^13^C-NMR (DMSO, 100 MHz): δ_C_ 164.3 (C-2), 103.3 (C-3), 182.1 (C-4), 161.9 (C-5), 99.2 (C-6), 164.5 (C-7), 94.2 (C-8), 157.7 (C-9), 104.1 (C-10), 121.9 (C-1′), 113.7 (C-2′), 146 (C-3′), 150 (C-4′), 116 (C-5′), 119.4 (C-6′).

***Compound 3*** was obtained as a white amorphous powder (7 mg)

^1^H-NMR (MeOD, 400 MHz): *δ*_H_ 5.11 (1H, d, *J*=Hz, H-1), 6.33 (1 H,d, *J* = 6 HZ, H-3), 5.12 (1H, dd, *J* = 6.1, 3.9 Hz, H-4), 2.89 (1H, m, H-5), 4.45 (1H, m, H-6), 5.78 (1H,s, H-7), 3.21 (1H, t, H-9), 4.3–4.8 (2H, d, *J* = 15 Hz, H-10), 4.70 (1H, d, *J* = 7.8 Hz, H-1′), 3.21 (1 H, d, *J*=Hz, H-2′), 3.37 (1H, t, H-3′), 3.30 (1H, t, H-4′), 3.33 (1H, m, H-5′), 3.86 (1H, d, *J* = 2 Hz, H6′A), 3.67 (1H, d, *J* = 2 Hz, H6′B).

^13^C-NMR (MeOD, 100 MHz): *δ*_C_ 96.3 (C-1), 140.2 (C-3), 104.4 (C-4), 46.5 (C-5), 81.4 (C-6), 128.9 (C-7), 146.6 (C-8), 47.8 (C-9), 61.2 (C-10), 100 (C-1′), 73.5 (C-2′), 76.8 (C-3′), 70.2 (C-4′), 76.4 (C-5′), 60 (C-6′).

***Compound 4*** was obtained as a buff powder (7 mg).

^1^H-NMR (DMSO, 400 MHz): *δ*_H_ (H1, *δ*6.91), (H2, *δ*9.19), (H3, *δ*8.85), (H4, *δ*12.24). ^13^C-NMR (DMSO, 100 MHz): *δ*_C_ 120.4 (C-1), 108.7 (C-2, C-6), 137.9 (C-3, C-5), 136.6 (C-4), 167.4 (C-7).

#### HPLC analysis

The HPLC profiling was carried out to identify flavonoid and phenolic compounds in the MeOH extract. Compounds were identified by comparing peak retention times and absorption spectra of unknown peaks with reference standards provided from Central Lab, Chromatography Analysis Unit, National Research Center, Giza, Egypt.

The methanol extract (5 g) was extracted with aqueous acetone (70%, 100 mL) using an Ultra-Turrax blender. After removing the acetone, the residue (3 g) was sonicated in 3 mL of methanol (5 min) then centrifuged at 1000 rpm (10 min). The supernatant was filtered through a 0.2-millipore membrane filter before HPLC analysis. Separation and determination of phenolics were performed using Hewlett Packard HPLC system (series 1050) equipped with an autosampling injector, a solvent degasser, a quaternary HP pump (series 1050), a Lichrosorb RP-18 column (4.0 mm i.d., 250 mm; 5 μm) (Merck, Darmstadt) and an ultraviolet (UV) detector set at (280 and 330 nm for phenolics and flavonoids, respectively). The column temperature was maintained at room temperature. Elution was carried out using methanol and acetonitrile (2:1) as a mobile phase at flow rate of 1 mL/min. Peak assignment was confirmed by injection of authentic phenolics and flavonoids. The retention time and peak area were used to calculate compound concentrations by the data analysis of Hewlett Packard software. The relative concentrations of the detected compounds were determined from the peak areas.

#### *In vitro* antioxidant activity

*In vitro* antioxidant activity of the MeOH and *n*-BuOH extracts of *V. nubicum* were evaluated using 1,1-diphenyl-2-picrylhydrazyl radical (DPPH·), 2,2′-azinobis (3-ethylbenzo-thiazoline-6-sulfonic acid) radical (ABTS·^+^), H_2_O_2_ and superoxide assays.

##### DPPH assay

The procedure of Hamauzu et al. (2005) was followed to determine radical scavenging activity using DPPH assay. DPPH (0.1 mM) solution in methanol was prepared and 4 mL was added to 0.2 mL of Trolox as well as MeOH and *n*-BuOH extracts of *V. nubicum* at different concentrations (50–250 µg/mL). The decrease in absorbance at 517 nm was measured at 60 min. A control test was carried out using 0.2 mL of distilled water instead of MeOH and *n*-BuOH extracts.

##### ABTS^•+^ radical assay

The ABTS^•+^ decolorization assay was performed using the procedure of Re et al. (1999). The ABTS^•+^ radicals were generated by the oxidation of ABTS (7 mM) in deinonized water with potassium persulfate (2.45 mM) mixed in ratio of 2:1 and kept in the dark at room temperature for 16 h. The ABTS^•+^ solution was diluted with ethanol to obtain absorbance of 0.700 ± 0.020 at 734 nm. ABTS^•+^ (3 mL) solution was mixed with Trolox (standard antioxidant drug) as well as MeOH and *n*-BuOH extracts of *V. nubicum* (0.1–0.8 mg/mL), incubated at room temperature for 6 min and their absorbance was recorded at 734 nm. Blanks were run in each assay. The inhibition percentage calculated using the following formula:

(1) $$\text{Inhibition}\;\text{percentage}\;\left(\%\right)=[(Ao-A_{1})/A_{1}]\times 100$$

where *A*_0_ is the absorbance of the control, and *A*_1_ is the absorbance of the sample.

##### H_2_O_2_ assay

The procedure of Guddadarangavvanahally et al. (2004) was used for H_2_O_2_ scavenging activity assay. A 2-mL aliquot of H_2_O_2_ (20 mM) solution prepared in PBS (pH 7.4) was added to 1 mL (100 μg/mL) of extracts and/or standard antioxidant drugs (BHT and α-tocopherol) then incubated for 10 min. The absorbance of the solution was measured at 230 nm against a blank solution containing MeOH and *n-*BuOH extracts without H_2_O_2_.

##### Superoxide assay

Superoxide radical assay was determined by the method of Elizabeth and Rao (1990). The reduction of nitroblue terazolium (NBT) by superoxide was measured in the presence and absence of MeOH and *n*-BuOH extracts of *V. nubicum*. The assay mixture included 0.1 mL of NBT (0.1 mg) and 0.3 mL with concentration (100 mg/L) of the extracts and/or Trolox as standard antioxidant drug and 1 mL of alkaline dimethyl sulfoxide (DMSO) in a final volume of 1.4 mL. Absorbance was measured at 560 nm.

### *In vivo* biological studies

#### LD_50_ estimation

##### Animals

Male albino rats weighing around 150 ± 10 g (240 rats; 120 for LD_50_ estimation, 36 for antiulcerogenic activity, 36 for anti-inflammatory and antinociceptive activities and 48 for hepatoprotecive activity) were obtained from the animal house of Faculty of Veterinary Medicine, Cairo University, Giza, Egypt. They were housed in plastic cages with stainless steel covers at the National Cancer Institute Animal House. The animals were maintained at a temperature of 22 ± 1 °C and a humidity of 55–60% in a light-controlled room. The animals were kept for 1 week to acclimatize, and then they were divided into 12 groups (*n* = 10). Animals were provided with standard diet and water *ad libitum*. All adopted procedures were in accordance with the guidelines of the AMS Directive 2018; 18/001/AMS for animal experiments and were approved by the Institutional Research Ethics Committee at the Faculty of Applied Medical Sciences, October 6 University, Egypt (No. 20180901).

##### Determination of LD_50_ of MeOH and n-BuOH extracts of *V. nubicum*

Preliminary experiments were carried out on six groups of four rats. MeOH and *n-*BuOH extracts of *V. nubicum* were orally administrated in different doses to find out the range of doses which cause 0 and 100% mortality of animals. A range doses was determined for each extract.

LD_50_ was determined by oral administration of MeOH extract in different doses 4000, 5000, 7500, 9000, 10,500 and 12,000 mg/kg. In group of 10 animals each, butanol extract was given orally in doses of 3000, 4000, 4500, 5000, 6000 and 7000 mg/kg.

After administration of the tested MeOH and *n-*BuOH extracts of *V. nubicum*, animals were observed individually every hour during the first day and every day for 21 days. Behavior and clinical symptoms of animals were noted throughout the duration of the experiment. The LD_50_ was calculated by Finney (1964) method by the application of the following formula:

(2) $${\text{LD}}_{\text{5}0}\;=\;\text{Dm}\;–\;\sum (z\;\times \;d)/n$$

Dm = The largest that kill all animals.

∑ = The sum of (*z* × *d*).

*z* = Mean of dead animals between two successive groups.

*d* = The constant factor between two successive doses.

*n* = Number of animals in each group.

##### Antiulcerogenic activity of MeOH and n-BuOH extracts of *V. nubicum*

###### Experimental design

The rats were randomized and divided into six groups of six rats each. Food was withdrawn 24 h and water 2 h before the commencement of experiment (Nwafor et al. 2000). The animals were divided into the following groups:

**Group I** received 1 mL saline solution orally, and kept as control group.
**Group II** was administered with 100 mg/kg of indomethacin.
**Groups III and IV** were pretreated with 200 and 400 mg/kg of the MeOH extract.
**Groups V and VI** were pretreated with 100 and 200 mg/kg of *n*-BuOH extract.

One hour prior to administration of 100 mg/kg of indomethacin, the extracts were administered intragastrically via the aid of an orogastric cannula. Four hours later, the animals were killed after light anaesthesia; the stomachs were removed and opened and its contents drained into a measuring cylinder. The pH of the contents was measured with a digital pH meter (PICO, Labindia Instruments Private Limited).

Macroscopic examination was carried out with a hand lens and scored for the presence of lesions (Nwafor et al. 2000).

Ulcer index (UI) and preventive ratio of each of the groups pretreated with extracts were calculated using the following formulae:

(3) UI=[degree of ulceration×percentage of group ulcerated]/100

Preventive ratio (%)

(4) (PO)=[(Ulcerated group – Protected group)×100]/Ulcerated group

(5) Degree of ulceration=total ulcer score/number of ulcerated animals

To determine the concentration of acid, the gastric contents were centrifuged at 1000 rpm for 10 min, 10 mL of gastric juice sample from the stomach of the animal was pipetted into a 250-mL conical flask 2–3 drops of phenolphthalein indicator was added and titrated against 0.1 M NaOH until a faint pink color is obtained. The value obtained was used to measure the total acidity using the formula below (Kulkarni et al. 2007) and expressed as in Equation (6).

(6) Total acidity=Volume of NaOH×normality of NaOH×100/0.1M  (Eq/L)

##### Anti-inflammatory and antinociceptive activities

The anti-inflammatory activity was carried out following the method of Domenjoz (1952). Rats (150–160 g) were divided into six different groups each of six animals (one control and five treatment groups). In the beginning, the thickness of the left paw was measured. They were treated orally with the MeOH (200 and 400 mg/kg b.w.) and *n*-BuOH (100 and 200 mg/kg b.w.) extracts of *V. nubicum* as well as indomethacin 60 mg/kg as a reference standard. After 30 min of administration, the inflammation was induced by s.c. injection of 0.1 mL of 6% formalin solution in normal saline. The right hind paw was injected with an equal volume of saline. The difference in thickness between the two paws gave the swelling induced by formalin. The anti-inflammatory efficacy was estimated by comparing the swelling of the treated with the control.

The Linear diameter of the injected paw was measured (with a transparent millimeter ruler) for 2 h at 30 min intervals after the administration of the formalin solution. Increases in the linear diameter of the right hind paws were taken as indicators of paw edema. Edema was assessed in terms of the difference in the ‘zero time’ (*Co*) linear diameter of the injected right hind paw, and its linear diameter at ‘time t’ [(*Ct*) – that is, 30, 60, 90 and 120 min] following formalin solution administration. The increases in the right hind paw diameters induced by injections of indomethacin were compared with those of the contra-lateral, non-injected left hind paw diameters (Muko and Ohiri 2000). Rats in the reference, comparative ‘test’ Group IV received indomethacin (60 mg/kg b.w.); while rats in the ‘control’ Group I received distilled water (3 mL/kg b.w.) only. Percentage inflammation (edema) was calculated from the formula: *Co*/*Ct* × 100; while percentage inhibition of the edema was calculated from the formula: *Co* − *Ct*/*Co* × 100 [where Co is the average inflammation (hind paw edema) of the ‘control’ Group I rats at a given time; and Ct is the average inflammation of the (Groups II–V) plant’s extract or (Group IV) indomethacin-treated rats at the same time].

The antinociceptive effect of the MeOH and *n*-BuOH of *V. nubicum* extracts was investigated in rats using the method described by Koster et al. (1959). Six groups of eight rats (150 ± 10 g) each were used for the controls and treated rats. The MeOH (200 and 400 mg/kg b.w.) and *n*-BuOH (100 and 200 mg/kg b.w.) extracts of *V. nubicum*, standard analgesic drug aspirin (100 mg/kg) or control vehicle were orally administrated 30 min before acetic acid (0.6%). Acetic acid was administrated intraperitoneally to all rats at the dose of 10 mL/kg and the number of abdominal constrictions with stretching of the hind limbs was counted over a period of 30 min as previously reported (Korster et al. 1959; Reanmogkol et al. 2000). The percentage of inhibition was expressed as a percentage of reduction of the number of abdominal contractions in treated animals with respect to the control.

##### Hepatoprotective effect of MeOH and n-BuOH extracts of *V. nubicum* in LPS-induced liver toxicity in rats

###### Experimental design

Animals were randomly assigned to six groups, eight rats in each.

**Group I:** received 1 mL normal saline orally and kept as normal control.
**Group II:** received LPS at a single i.p. dose (10 mg/kg b.w.) and kept as a positive control.
**Groups III and IV**: were treated with MeOH extract at a dose of (200 and 400 mg/kg b.w.), suspended in normal saline orally in a single daily dose for 10 days followed by a single i.p. dose of LPS (10 mg/kg b.w.).
**Groups V and VI:** were treated with *n*-BuOH extract at a dose of (100 and 200 mg/kg b.w.) suspended in normal saline orally in a single daily dose for 10 days followed by a single i.p. dose of LPS (10 mg/kg b.w.).

On 11th day, blood was collected from the retro-orbital vein of each animal and each sample was collected into two heparinized tubes. The first part of heparinized blood samples was divided into two aliquots.

The first aliquot was used for determination of GPx and CAT activity according to the methods of Paglia and Valentine (1967) and Sinha (1972), respectively.

The second aliquot was hemolyzed using double distilled water and the hemolysate of each sample was divided into two portions. The first portion was precipitated with chloroform/ethanol (3:5 V/V) mixture and the resultant supernatant was used for the determination of SOD activity according to the methods of Marklund and Marklund (1974).

The second portion was deproteinized with *meta*'-phosphoric acid and the clear supernatant was used for the estimation of GSH level (Sedlak and Lindsay 1968). Hemoglobin and white blood cells count were determined in the heparinized blood samples and used in the calculation of the enzyme activity (CAT and SOD) as well as GSH level (Van Kampen and Zijlstra 1961).

The second part of the heparinized blood samples was allowed to centrifuge at 1000 *g* for 20 min. The separated plasma was used for the estimation of serum activity of ALT, AST, and LDH according to the methods of Reitman and Frankel (1957) and King (1965).

Rats were sacrificed by cervical dislocation after light anesthesia and placed on disposable pads abdomen up and the liver was excised immediately and washed off from blood with ice-cold physiological saline. Then, the tissue was blotted in between filter papers to absorb moisture. A 10% organ homogenate was prepared in 0.1 M Tris-HCl buffer (pH 7.4). The homogenate was centrifuged at 3000 rpm for 15 min and the supernatant was used for the estimation of liver activity of SOD, CAT, and GPx according to the reported methods (Paglia and Valentine 1967; Sinha 1972; Marklund and Marklund 1974). Also, levels of GSH, TNF-α, NO, TBARS and total protein in liver homogenate were estimated according to the methods previously described (Sedlak and Lindsay 1968; Nichans and Samulelson 1968; Beyaert and Fiers 1998; Miranda et al. 2001).

### Isolation of peritoneal macrophages

Rats were sacrificed and placed on disposable pads abdomen up, the skin was carefully cut by scissors above the peritoneum about midway the length of the abdomen according to the method of Kolaczkowska et al. (2008) and peritoneal macrophages were isolated as follows. Through the exposed peritoneal membrane, 10 mL of cold sterile PBS (Invitrogen/Gibco) was injected into the peritoneal cavity by a sterile syringe and 22-gauge needle. The peritoneal cavity was gently massaged to loosen the macrophages; the enriched fluid was collected by the same syringe into a 50-mL Falcon tube and centrifuged (10 min, 200 *g*, 4 °C). Cells were washed with complete medium and counted using hemocytometer. The number of viable cells was calculated and kept constant at 1 × 10^6^ cell/mL.

### Determination of NO and PGE_2_ production

Diluted cell suspensions of peritoneal macrophages (5 × 10^5^ cells/mL), plated onto 48-well plates and were pre-incubated for 2 h. Then, the MeOH and *n*-BuOH extracts at doses of (50, 100 and 200 µg/mL) were added to the cells and later treated with LPS (1 µg/mL) at 37 °C for 18 h.

The level of NO production was determined by assaying the culture supernatants for nitrite, which is the stable product of a reaction between NO and molecular oxygen, using the Griess reagent, as described previously (Chu et al. 2016).

PGE_2_ levels were measured using diluted cell suspensions of peritoneal macrophages (1 × 10^5^ cells/well) that were incubated with the MeOH and *n*-BuOH extracts (50, 100 and 200 µg/mL) and later treated with LPS (1 µg/mL) for 18 h. The PGE_2_ level in the supernatants was estimated using a specific enzyme immunoassay kit (Cayman Chemicals, San Diego, CA, USA).

### Measurement of COX-2 enzyme activity

COX-2 enzyme activity was measured using a fluorescent activity assay kit (Cayman Chemicals, San Diego, CA, USA), according to the manufacturer’s specifications. Briefly, diluted cell suspensions of peritoneal macrophages (1 × 10^5^ cells/well) were incubated with MeOH and *n*-BuOH extracts (50, 100 and 200 µg/mL). Then wells were cultured with the vehicle and test extracts in the presence of 1 µg/mL of LPS for 18 h.

### Measurement of TNF-α production

Diluted cell suspensions of peritoneal macrophages (1 × 10^6^ cells/well) in 48-well plates were treated with MeOH and *n*-BuOH extracts (50, 100 and 200 µg/mL) and later treated with LPS (1 µg/mL) at 37 °C for 18 h. The supernatants were subsequently employed for the pro-inflammatory cytokine (TNF-α) assay using a mouse enzyme-linked immunosorbent assay (ELISA) kit (R&D Systems, Minneapolis, MN, USA).

#### Histopathological examination

Liver and stomach were sliced, and pieces were preserved in 10% formalin for proper fixation. These tissues were processed and embedded in paraffin wax. Sections of 5–6 microns in thickness were cut and stained with staining reagents. All the sections of the tissues were examined under microscope according to the method of Bancroft and Steven (1983).

### Statistical analysis

The results are expressed as means *±* SD. Comparisons between groups were performed using a one-way analysis of variance (ANOVA). Differences between individual treatment groups were compared using Dunnett’s test. Statistical significance was set at *p* < 0.05 and *p* < 0.01, and the statistical analyses were performed using SPSS software, version 15.0 (SPSS, Inc., Chicago, IL, USA).

## Results

**Figure 1.**
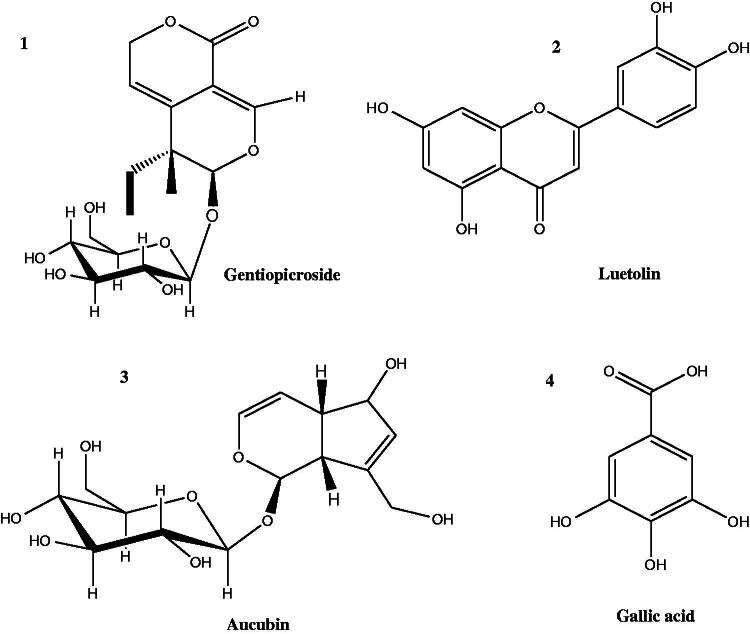
Chemical compounds isolated from *Verbascum nubicum*.

Four compounds **1–4** were isolated from the aerial parts of the *n*-BuOH extract of *V. nubicum* and identified as gentiopicroside, luteolin, aucubin, and gallic acid, respectively, using NMR data (^1^H- and ^13^C-NMR), they were confirmed by comparison with reported data (Tatli and Akdemir 2004; Mahmoud et al. 2007) and, co-chromatography with available standards. Structures of the isolated compounds are represented in Figure 1.

### HPLC profiling

HPLC profiling of MeOH extract was performed by comparing retention times and absorption spectra of the unknown peaks with reference standards. HPLC analyses showed that luteolin 7-glucoside (2215.43 mg/100 g) was the most dominant compound, followed by hesperidin (954.51 mg/100 g) and apigenin (233.15 mg/100 g). Results are compiled in Table 1.

**Table 1.** HPLC of identified Flavonoids in the MeOH extract of *Verbascum nubicum*.

Flavonoids identified	Conc. (mg/100 g)
Apigenin 6-*O*-arbinoside 8-*O*-glactoside	26.81
Apigenin 6-*O*-rhamnoside 8-*O*-glucoside	10.79
Naringin	67.13
Luteolin 7-glucoside	2215.43
Hesperidin	954.51
Rosmarinic	108.74
Apigenin 7-glucoside	207.61
Apigenin 7-*O*- neohespiroside	63.04
Kampferol 3,7-dirhamoside	28.14
Quercetrin	74.44
Kampferol 3-(2-*p*-coumaroyl) glucoside	134.58
Naringenin	70.35
Acacetin 7-neohesperidoside	211.25
Hesperitin	91.04
Kampferol	202.75
Apigenin	233.15

The major identified phenolics were gallic acid (988.1 mg/100g) followed by ellagic acid (793.81 mg/100 g) and catchein (313 mg/100 g). Results are compiled in Table 2.

**Table 2.** HPLC of identified Phenolics in MeOH extract of *Verbascum nubicum*.

Phenolics identified	Conc. (mg/100 g)
Gallic	988.1
Pyrogallol	12.87
3-OH Tyrosol	23.64
Protocatchuic	14.56
Catechein	313.33
Catechol	256.42
*p*-OH-benzoic	17.66
Caffeic acid	84.47
Vanillic	266.48
*p*-Coumaric	15.68
Ferulic	17.44
*iso*-Ferulic	18.42
*o*-coumaric	20.76
Ellagic	793.81
Coumarin	13.18
3,4,5-methoxy-cinnamic	26.56
Cinnamic	11.84

### Biological studies

#### *In vitro* antioxidant activity

**Figure 2.**
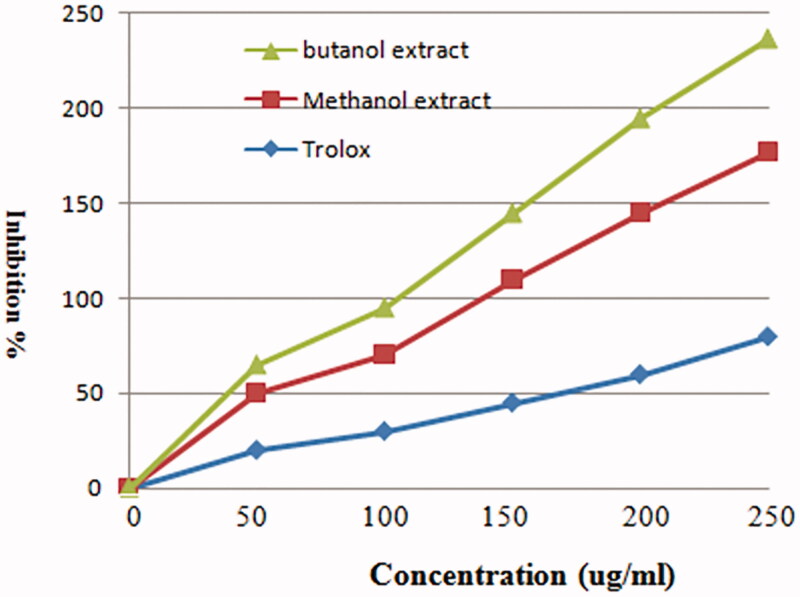
Scavenging activities of different concentrations of MeOH and *n*-BuOH extracts of *Verbascum nubicum* and Trolox against the 1,1-diphenyl-2-picryl-hydrazil (DPPH**^·^**) radical. *In vitro* antioxidant activity of methanol and butanol extracts of *Verbascum nubicum*.

**Figure 3.**
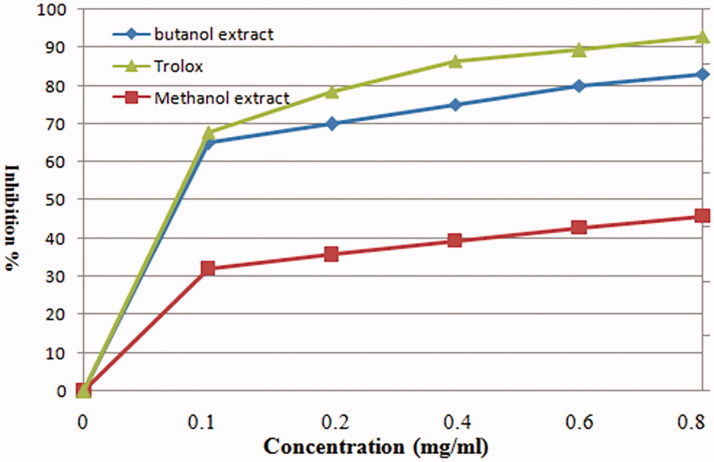
Scavenging activities of different concentrations of MeOH and *n*-BuOH extracts of *V. nubicum* and Trolox against ABTS^•+^ radical.

Figure 2 shows the scavenging activity of MeOH and *n*-BuOH extracts of *V. nubicum* at different concentrations and Trolox as the reference standard against the 1,1-diphenyl-2-picryl-hydrazil (DPPH**^·^**) radical. MeOH and *n*-BuOH extracts at 250 μg/mL had the highest radical scavenging activity when compared with Trolox. In addition, the ABTS^•+^ radical scavenging effects of MeOH and *n*-BuOH extracts are presented in Figure 3 and showed appreciable free radical scavenging activities. The free radical scavenging activity of MeOH and *n*-BuOH extracts was comparable to Trolox. The *n*-BuOH extract at a concentration of 0.8 mg/mL had the highest radical scavenging activity when compared with MeOH extract. Radical scavenging activity of MeOH and *n*-BuOH extracts and Trolox is arranged in the following order: Trolox > *n*-BuOH extract > MeOH extract.

**Figure 4.**
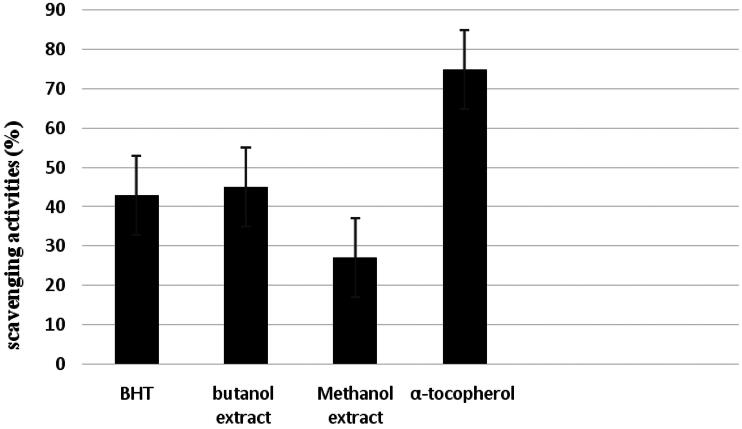
Hydrogen peroxide scavenging activity of MeOH and *n*-BuOH extracts of *V. nubicum*, α-tocopherol and BHT at 100 μg/mL concentration.

Figure 4 presents the scavenging activity of the tested samples on H_2_O_2_. The results are compared with BHT and α-tocopherol as standards. At 100 µg/mL, *n*-BuOH and MeOH extracts exhibited 54.0 and 27.0% inhibition, respectively. Tocopherol and BHT exhibited inhibition of 75.0 and 43.0%, respectively, of H_2_O_2_ scavenging activity at the same concentration.

**Figure 5.**
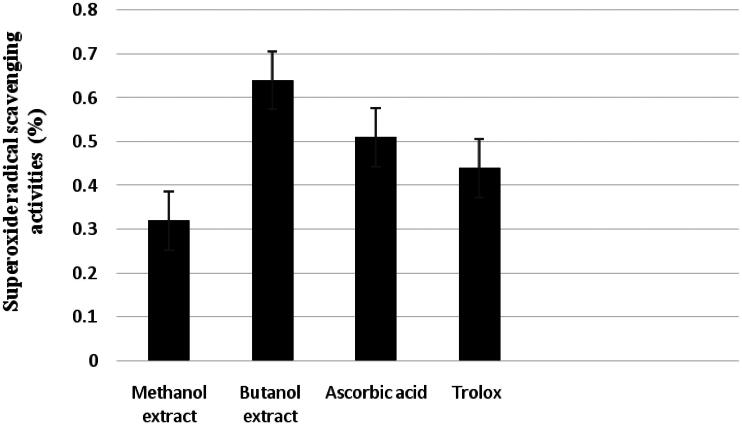
Superoxide radical scavenging activity of MeOH and *n*-BuOH extracts of *Verbascum nubicum* and Trolox at 100 mg/L concentration.

Figure 5 shows the superoxide radical scavenging activity at 100 mg/L of MeOH and *n*-BuOH extracts in comparison to the same amount of Trolox. At 100 mg/L concentrations, *n*-BuOH extract showed a higher superoxide radical scavenging activity than Trolox and MeOH extract. The superoxide radical scavenging activity of MeOH, *n*-BuOH and both standards decreased in the following order: *n*-BuOH extract > ascorbic acid > Trolox > MeOH extract.

#### Determination of LD_50_ of methanol and butanol extracts of *V. nubicum* in adult rats

From the equation, the LD_50_ of MeOH and *n*-BuOH extracts were calculated to be 8200 and 4225 mg/kg b.w., respectively.

Observation of animals received both extracts showed an increase in heart rate, rapid respiration within 1–2 h. There is a general depression in activity with tremors in hind limbs. The mucous of the eye became brownish in color and the skin and toes bluish. The temperature of the animals’ extremities dropped with the toes and tail being cool.

#### Effect of MeOH and n-BuOH extracts of *V. nubicum* on indomethacin-Induced ulceration in rats

As indicated in Table 3, administration of indomethacin resulted in the production of gastric lesions. The mean gastric ulcer index for this group (group II) was 27.54 ± 3.60 which is significantly higher (*p* < 0.01) than for the control (0.54 ± 0.17). The MeOH extract of *V. nubicum* at 200 and 400 mg/kg b.w. lowered (*p* < 0.01) the index for the indomethacin-induced ulcer to 13.38 ± 1.70 and 9.60 ± 2.00, respectively. Also, the *n*-BuOH extract (100 and 200 mg/kg b.w.) lowered (*p* < 0.01) the index for the indomethacin-induced ulcer to 11.84 ± 2.30 and 4.25 ± 0.63, respectively. In addition, administration of indomethacin resulted in the elevation of the acid concentration in gastric contents. The mean level of acid in gastric content for group II was 0.35 ± 0.06, which is significantly higher (*p* < 0.01) than for the control group (0.15 ± 0.03). The MeOH extract (200 and 400 mg/kg b.w.) lowered (*p* < 0.01) the level of acid in gastric content for an indomethacin-induced ulcer to 0.27 ± 0.04 and 0.15 ± 0.04, respectively. However, the *n*-BuOH extract of *V. nubicum* (100 and 200 mg/kg b.w.) lowered (*p* < 0.01) the level of acid in gastric content for indomethacin-induced ulcer to 0.19 ± 0.02 and 0.09 ± 0.01, respectively. In addition, administration of indomethacin resulted in depletion of gastric pH. The mean pH of gastric content for group II was 2.30 ± 0.32, which is significantly lower (*p* < 0.01) than for the control group (2.93 ± 0.42). The MeOH extract of *V. nubicum* at (200 and 400 mg/kg b.w.) increased (*p* < 0.01) the gastric pH for indomethacin-induced ulcer to 3.10 ± 0.43 and 3.40 ± 0.20, respectively. The *n*-BuOH extract of *V. nubicum* at 100 and 200 mg/kg b.w. increased (*p* < 0.01) the level of gastric pH for the indomethacin-induced ulcer to 3.65 ± 0.40 and 3.80 ± 0.34, respectively.

**Table 3.** Effect of MeOH and *n*-BuOH extracts of *Verbascum nubicum* on gastric acidity parameters and ulcer index on indomethacin-induced ulceration in rats.

Groups	Treatment (mg/kg)	Ulcer index (UI)	Protection (%)	Concentration of acid in gastric contents (meq/L)	pH of gastric contents
I	Negative control (2 mL saline)	0.54 ± 0.17	–	0.15 ± 0.03	2.93 ± 0.42
II	Indomethacin (100 mg/kg b.w.)	27.54 ± 3.60	–	0.35 ± 0.06	2.30 ± 0.32^a^
III	MeOH extract (200 mg/kg b.w.)	13.38 ± 1.70^a^	51.42	0.27 ± 0.04^a^	3.10 ± 0.43^a^
IV	MeOH extract (400 mg/kg b.w.)	9.60 ± 2.00^a,b^	65.14	0.15 ± 0.04^a,b^	3.40 ± 0.20^a,b^
V	*n*-BuOH extract (100 mg/kg b.w.)	11.84 ± 2.30^a,b,c^	57.00	0.19 ± 0.02^a,b,c^	3.65 ± 0.40^a,b,c^
VI	*n*-BuOH extract (200 mg/kg b.w.)	4.25 ± 0.63^a,b,c,d^	84.57	0.09 ± 0.01^a,b,c,d^	3.80 ± 0.34^a,b,c,d^

Results are mean ± S.E.M, *n* = 6. ^a^*p* < 0.01 significant relative to group I; ^b^*p* < 0.01 significant relative to group II; ^c^*p* < 0.01 significant relative to group III and ^d^*p* < 0.01 significant relative to group IV.

**Figure 6.**
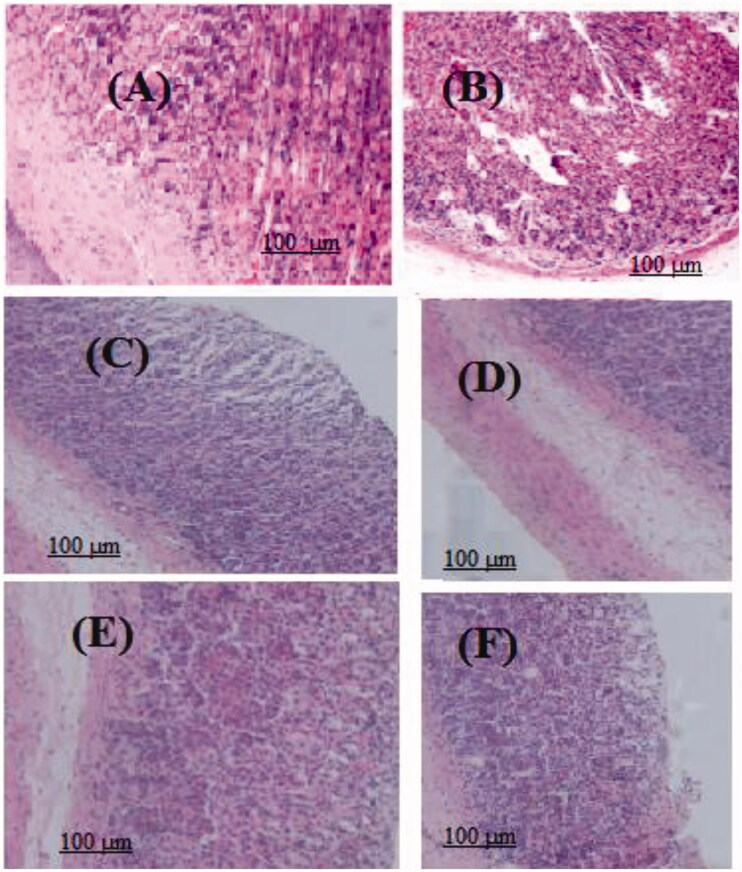
Sections stained with hematoxylin and eosin (H&E; 100×) displaying the regenerated glandular epithelium width in stomachs of rats treated with indomethacin. Also, antiulcerogenic activity of MeOH and *n*-BuOH extracts of *V. nubicum* on indomethacin-induced ulceration in rats. (A) Negative control (2 mL saline); (B) Indomethacin (100 mg/kg b.w.); (C) MeOH extract (200 mg/kg b.w.); (D) MeOH extract (400 mg/kg b.w.); (E) *n*-BuOH extract (100 mg/kg b.w.); (F) *n*-BuOH extract (200 mg/kg b.w.).

Histopathological sections showed that degeneration of glandular epithelium in indomethacin treated group II (Figure 6(B)) significantly increased (*p* < 0.01) than for the control (Figure 6(A)) Also, sections of ulcerated area revealed that there was a significant increase in regenerated glandular epithelium width after treatment with the MeOH extract 200 and 400 mg/kg b.w.) (Figure 6(C,D)). The collagen content in the ulcerated tissue was significantly increased by *n*-BuOH extract of both doses, showing the maximum effect (Figure 6(E,F)). No significant difference on capillary density in scar tissue was observed after treatment with both extracts (Figure 6(C–F)).

#### Anti-inflammatory and antinociceptive effects of MeOH and n-BuOH extracts of *V. nubicum*

Inflammation (edema) was always evident within 5–8 min following 6% formalin solution injection; maximal swelling and/or edema occurred approximately 120 min following 6% formalin solution administration. The MeOH and *n*-BuOH extracts also produced a significant reduction (*p* < 0.01) in the formalin solution-induced acute inflammation of the rat hind paw (Table 4).

**Table 4.** Anti-inflammatory activity of MeOH and *n*-BuOH extracts of *V. nubicum*.

		Formalin-induced rat paw edema thickness (mm)/min			
Groups	Treatment (mg/kg)	30	60	90	120
I		15.65 ± 0.15	14.80 ± 0.39	15.00 ± 0.29	15.50 ± 0.25
II	MeOH extract (200 mg/kg b.w.)	13.78 ± 0.09* (11.94%)	10.70 ± 0.24* (27.7%)	9.88 ± 0.32** (34.10%)	7.30 ± 0.15** (52.90%)
III	MeOH extract (400 mg/kg b.w.)	9.50 ± 0.08** (39.29%)	8.50 ± 0.14** (42.65%)	8.00 ± 0.048** (46.6%)	5.90 ± 0.051**** (61.93%)
1V	*n*-BuOH extract (100 mg/kg b.w.)	11.89 ± 0.06* (24.02%)	9.17 ± 0.06** (38.04%)	7.08 ± 0.05** (49.33%)	6.48 ± 0.066*** (58.19%)
V	*n*-BuOH extract (200 mg/kg b.w.)	9.30 ± 0.09** (40.37%)	8.00 ± 0.07** (45.94%)	7.60 ± 0.05** (49.33%)	6.80 ± 0.09*** (56.13%)
VI	Indomethacin (60 mg/kg b.w.)	7.25 ± 0.09** (53.67%)	6.80 ± 0.069*** (54.05%)	6.56 ± 0.071*** (56.26%)	5.80 ± 0.048*** (62.58%)

*Note*: Indomethacin is used as a reference. *Significant at *p* < 0.05; **Significant at *p* < 0.01; ***Significant at *p* < 0.005.

The MeOH and *n*-BuOH extracts of both doses induced a significant reduction in the number of contortions provoked by an i.p. injection of acetic acid in rats (Table 5). The maximum reduction of the writhing response of MeOH extract was 61.57% at the dose of 400 mg/kg. Also, the maximum reduction of the writhing response of *n*-BuOH extract was 66.46% at the dose of 200 mg/kg. The positive control drug, aspirin (100 mg/kg) also provoked significant protective effect (60.91%) against acetic acid-induced pain.

**Table 5.** Effect of MeOH and *n*-BuOH extracts of *V. nubicum* on acetic acid induced abdominal constriction in rats.

Groups	Treatment (mg/kg)	Number of writhings for 30 min	Inhibition (%)
I	Control	97.5 ± 3.5	
II	MeOH extract (200 mg/kg b.w.)	48.25 ± 4.10*	50.5%
III	MeOH extract (400 mg/kg b.w.)	37.47 ± 2.44*^a,b^	61.57%
IV	*n*-BuOH extract (100 mg/kg b.w.)	42.80 ± 2.16*^a^	56.10%
V	Butanol extract *n*-BuOH extract (200 mg/kg b.w.)	32.70 ± 3.00*^b^	66.46%
VI	Aspirin (100 mg/kg b.w.)	38.11 ± 2.40*^a^	60.91%

*Note*: Aspirin is used as a reference. *Significant at *p* < 0.05, Groups (II–VI) were compared with group (I). Data shown are mean ± standard deviation of number of observations within each treatment. Data followed by the same letter are not significantly different at *p* ≤ 0.05.

#### Hepatoprotective effect of MeOH and n-BuOH extracts *V. nubicum* in LPS-induced liver toxicity in rats

Tables 6 and 7 showed the enzymatic activity of plasma ALT, AST and LDH of control and experimental groups of rats. Activity of ALT, AST and LDH as well as TNF-α, NO, TBARS in plasma and liver homogenates was increased significantly in plasma of injured rats (group II) when injected with LPS (10 mg/kg) (*p* < 0.01) compared with the control group (I). Treatment of animals with MeOH and *n*-BuOH extracts of both doses improved the activity of these enzymes as well as TNF-α, NO and TBARS in plasma and liver homogenate significantly (*p* < 0.01) when compared with LPS-treated rats and in dose-depended manner. LPS (10 mg/kg), injection subcutaneously to all groups of rats markedly decreased GPx, CAT and SOD and GSH levels (*p* < 0.01) indicating acute hepatotoxicity compared with saline-treated ‘normal’ rats. Oral treatment with MeOH (200 and 400 mg/kg b.w.) and *n*-BuOH (100 and 200 mg/kg b.w.) of *Verbascum nubicum* significantly increase in the activities of these liver oxidative stress biomarker enzymes (*p* < 0.01) compared with LPS-treated rats. The effect was more pronounced in *n*-BuOH (200 mg/kg b.w.) (*p* < 0.01) compared to MeOH extract (*p* < 0.01).

**Table 6.** Effect of MeOH and *n*-BuOH extracts of *V. nubicumn* on oxidative stress biomarkers in plasma and liver homogenate.

No.	Groups	ALT (U/L)	AST (U/L)	LDH (U/L)	GPx (U/mL)	CAT (U/g Hb)	SOD (U/g Hb)	GSH (mg/g Hb)	TBARS (nmol/mL)
(I)	Normal 1 mL normal saline orally	12.76 ± 0.87	19.80 ± 2.11	136.25 ± 6.50	38.76 ± 3.25	83.60 ± 4.37	176.5 ± 6.55^@^	25.49 ± 2.59	2.87 ± 0.24
(II)	Control (LPS 10 mg/kg b.w.)	36.50 ± 2.60*	52.70 ± 3.44*	272.49 ± 11.25*	15.60 ± 2.55*	34.88 ± 9.04*	94.20 ± 5.32*	12.87 ± 1.35*	6.43 ± 0.98*
(III)	MeOH extract (200 mg/kg b.w.)+ LPS (10 mg/kg b.w.)	25.44 ± 1.38^@^	34.68 ± 5.00^@^	157.60 ± 10.90^@^	29.80 ± 5.43^@^	65.38 ± 4.50^@^	110.40 ± 12.65^@^	19.44 ± 1.62^@^	4.32 ± 0.32^@^
(IV)	MeOH extract (400 mg/kg b.w.)+ LPS (10 mg/kg b.w.)	20.54 ± 2.14^@^	29.44 ± 2.65^@^	147.28 ± 9.70^@^	36.70 ± 3.87^@^	85.34 ± 8.62^@^	154.63 ± 9.08^@^	24.33 ± 1.38^@^	3.76 ± 0.64^@^
(V)	*n*-BuOH extract (100 mg/kg b.w.)+ LPS (10 mg/kg b.w.)	22.10 ± 2.00^@^	35.48 ± 3.03^@^	165.64 ± 13.26^@^	26.60 ± 2.80^@^	56.70 ± 5.97^@^	105.25 ± 6.48^@^	20.90 ± 1.55^@^	4.10 ± 0.62^@^
(VI)	*n*-BuOH extract (200 mg/kg b.w.)+ LPS (10 mg/kg b.w.)	15.37 ± 1.20^@^	21.00 ± 2.87^@^	139.08 ± 8.74^@^	35.76 ± 4.07^@^	88.65 ± 6.04^@^	169.33 ± 8.74^@^	24.87 ± 2.16^@^	2.54 ± 0.32^@^

LPS was given i.p as a single dose of 10 mg/kg b.w. to 18 h fasted animals. It was given to all groups except the normal one. MeOH and *n*-BuOH extracts of *V. nubicum* were orally given daily for 10 days. Blood samples were collected 24 h after the last dose administration. Values are given as mean ± SD for groups of eight animals each. *Significantly different from normal group at *p <* 0.01; ^@^Significantly different from control group at *p <* 0.05.

**Table 7.** Activity of glutathione peroxidase (GPx), catalse (CAT), superoxide dismutase (SOD) as well as level of reduced glutathione (GSH), tumor necrosis factor-α (TNF-α), nitric oxide (NO) and thibarbaturic acid reactive substances (TBARs) in liver of normal and experimental groups of rats.

No.	Groups	GPx	CAT	SOD	GSH (mg/g tissue)	TNF-α (pg/g protein)	NO (Umol/g protein)	TBARS (nmol/g protein)
(I)	Normal 1 mL normal saline orally	7.09 ± 0.37	45.60 ± 3.78	11.35 ± 1.08^@^	5.43 ± 0.98	6.80 ± 0.43	9.43 ± 0.85	0.69 ± 0.07
(II)	Control (LPS 10 mg/kg b.w.)	2.69 ± 0.65*	18.65 ± 2.33*	3.67 ± 0.08*	2.11 ± 0.24*	22.54 ± 0.24*	36.54 ± 3.25*	1.54 ± 0.37*
(III)	MeOH extract (200 mg/kg b.w.) + LPS (10 mg/kg b.w.)	4.77 ± 0.55^@^	32.90 ± 3.22^@^	7.69 ± 0.98^@^	4.33 ± 0.31^@^	15.40 ± 1.65^@^	17.60 ± 1.79^@^	0.94 ± 0.086^@^
(IV)	MeOH extract (400 mg/kg b.w.) + LPS (10 mg/kg b.w.)	6.80 ± 0.60^@^	42.70 ± 2.65^@^	10.43 ± 1.25^@^	5.12 ± 0.87^@^	9.07 ± 0.54^@^	14.39 ± 2.66^@^	0.88 ± 0.054^@^
(V)	*n*-BuOH extract (100 mg/kg b.w.) + LPS (10 mg/kg b.w.)	6.11 ± 0.43^@^	33.17 ± 4.09^@^	9.00 ± 0.87^@^	3.65 ± 0.44^@^	19.26 ± 2.50^@^	26.50 ± 2.99^@^	0.75 ± 0.039^@^
(VI)	*n*-BuOH extract (200 mg/kg b.w.) + LPS (10 mg/kg b.w.)	7.25 ± 0.59^@^	44.76 ± 3.81^@^	12.45 ± 1.08^@^	5.74 ± 0.60^@^	9.80 ± 0.87^@^	11.60 ± 1.04^@^	0.65 ± 0.054^@^

LPS was given i.p as a single dose of 10 mg/kg.b.w. to 18 h fasted animals. It was given to all groups except the normal one. MeOH and *n*-BuOH extracts of *Verbascum nubicum* were orally given daily for 10 days. Liver samples were collected 24 h after the last dose administration. Values are given as mean ± SD for groups of eight animals each. *Significantly different from normal group at *p <* 0.01; ^@^Significantly different from control group at *p <* 0.05. SOD: one unit of activity was taken as the enzyme reaction, which gave 50% inhibition of NBT reduction in 1 min/mg protein; GPx: μg of GSH consumed/min mg protein; CAT: μmol of H_2_O_2_ utilized/min mg protein.

##### Histopathology examination of rat liver tissue in different experimental groups

**Figure 7.**
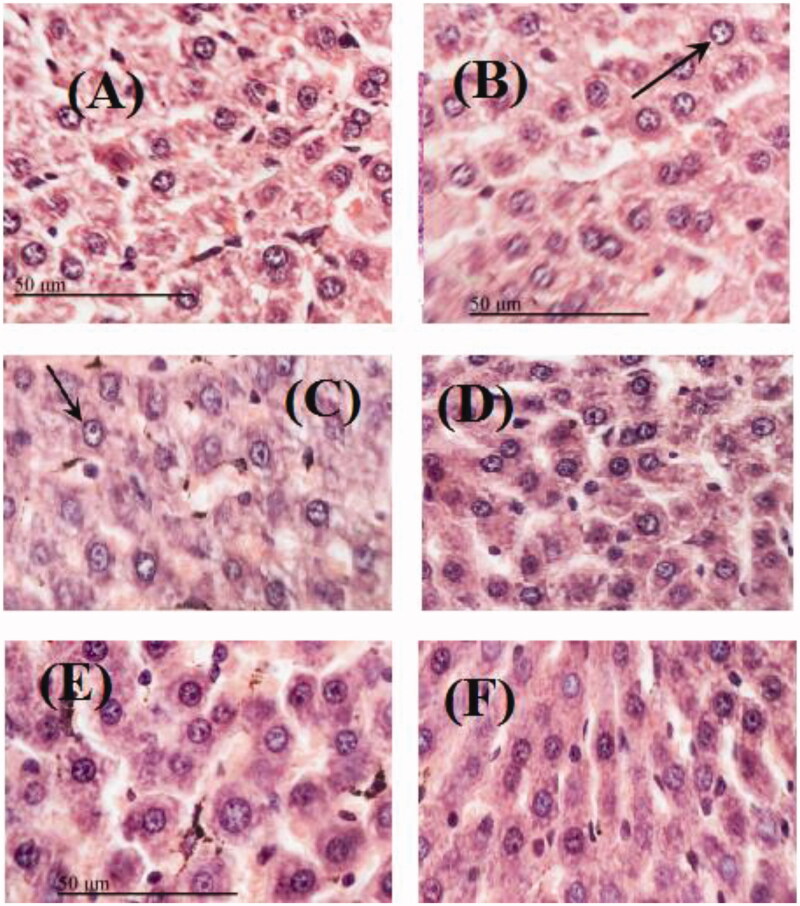
Sections stained with hematoxylin and eosin (H&E; 100×) histological examination of rats hepatocytes of different groups compared to control group (A); (B) LPS (10 mg/kg.B.W.); (C) MeOH extract (200 mg/kg b.w.); (D) MeOH extract (400 mg/kg.b.w.); (E) *n*-BuOH extract (100 mg/kg b.w.); (F) *n*-BuOH extract (200 mg/kg b.w.).

The control group (Figure 7(A)) had normal hepatocytes architecture showing normal portal vein, bile duct and hepatic artery. Rats injected with LPS (10 mg/kg b.w.) revealed the highest hepatocytes damage with inflammatory cells infiltration with many pigmented macrophages and brown granular deposits (Figure 7(B)). Liver sections of rats treated with MeOH extract of both doses (200 and 400 mg/kg b.w.) showed apparent normal histological structures of hepatic tissue. Animals of both groups of rats treated with *n-*BuOH extract (100 and 200 mg/kg b.w.) showed apparent normal organized hepatocytes with intact cellular details, normal blood vessels and minimal inflammatory cells infiltrations (Figure 7(E,F)).

The effects of *V. nubicum* extracts on the levels of inflammatory mediators were shown in Table 8. The data showed that the levels of peritoneal macrophage cells (PMCs), tumor necrosis factor-α (TNF-α), nitric oxide (NO), prostaglandin E2 (PGE_2_) as well as cyclooxygenase (COX-2) enzyme in the peritoneal macrophages cells were increased significantly in LPS-treated group (**II)** (1 µg/mL) (*P* < 0.01) compared within cells of the normal control group (**I)**. The treatment of the peritoneal macrophage cells injected with LPS (1 µg/mL) with both extracts of *V. nubicum* (50, 100 and 200 µg/mL) reduced the levels of PMCs, WBCs, TNF-α, PGE_2_ and COX-2 significantly (*p* < 0.01) compared with LPS-treated cells group (**II**) and in a dose-dependent manner. Level of WBCs in peritoneal macrophage cells was decreased significantly in peritoneal macrophage cells (group **II**) injected with LPS (1 µg/mL) (*p* < 0.01) compared with normal control group (**I**). Treating peritoneal macrophage cells with MeOH (50, 100 and 200 µg/mL) and *n*-BuOH (50, 100 and 200 µg/mL) elevated the levels of WBCs in peritoneal macrophage cells significantly (*p* < 0.01) compared with LPS-treated cells and dose dependent manner.

**Table 8.** Levels of peritoneal macrophage cells/mL (PMCs) and White blood cells (WBCs), tumor necrosis factor-α (TNF-α), nitric oxide (NO) and prostaglandin E2 (PGE_2_) as well as cyclooxygenase-2 (COX-2) enzyme activity in peritoneal macrophages of negative control and experimental groups.

No.	Groups	No. of PMCs,×10^6^	WBCs × 10^3^	TNF-α (pg/mL PM)	NO (Umol/mL PM)	PGE_2_ (pg/mL PM)×10^3^	COX-2 (U/mL PM)
I	Negative control	1.5 ± 0.14	4.75 ± 0.63	5.36 ± 0.48^@^	2.67 ± 0.17	0.54 ± 0.32	18.43 ± 1.65
II	Control (LPS 1 μg/mL)	3.6 ± 0.48*	2.50 ± 0.37*	13.90 ± 1.25*	5.78 ± 0.33*	2.65 ± 0.43*	84.50 ± 3.70*
III	MeOH extract (50 µg/mL) + LPS (1 µg/mL)	2.4 ± 0.36^@^	3.60 ± 0.76^@^	8.70 ± 0.63^@^	3.18 ± 0.21^@^	1.74 ± 0.35^@^	60.43 ± 5.09^@^
IV	MeOH extract (100 µg/mL) + LPS (1 µg/mL)	2.0 ± 0.44^@^	4.10 ± 0.46^@^	6.44 ± 0.50^@^	2.98 ± 0.48^@^	0.96 ± 0.075^@^	30.50 ± 3.25^@^
V	MeOH extract (200 µg/L) + LPS (1 µg/mL)	1.46 ± 0.35^@^	4.50 ± 0.17^@^	5.40 ± 0.43^@^	2.50 ± 0.27^@^	0.51 ± 0.086^@^	22.73 ± 2.60
VI	*n*-BuOH extract (50 µg/mL) + LPS (1 µg/mL)	2.14 ± 0.18^@^	4.00 ± 0.49^@^	7.63 ± 0.61^@^	2.85 ± 0.36^@^	1.45 ± 0.18^@^	57.20 ± 4.31^@^
VII	*n*-BuOH extract (100 µg/mL) + LPS (1 µg/mL)	1.9 ± 0.09^@^	4.90 ± 0.6^@.^	5.20 ± 0.46^@^	2.78 ± 0.22^@^	0.87 ± 0.051^@^	26.84 ± 4.17^@^
VIII	*n*-BuOH extract (200 µg/mL + LPS (1 µg/mL)	1.49 ± 0.09^@^	4.60 ± 0.40^@.^	5.40 ± 0.80^@^	2.70 ± 0.54^@^	0.48 ± 0.034^@^	19.32 ± 1.55^@^

LPS was added as a single dose of 1 µg/mL. It was added to all groups except the normal one. Data are presented as means *±* SD. *Significantly different from negative control group at *p < 0.01*; ^@^*p* < 0.01 compared to the LPS-stimulated group (ANOVA followed Dunnett’s test).

Antioxidant properties of MeOH and *n*-BuOH extracts of *V. nubicum* have been determined chemically by monitoring their capacity to scavenge the stable free-radical DPPH^•^, ABTS^•+^, H_2_O_2_ and superoxide. Assessment of their antioxidant activities revealed that *n*-BuOH extract was the most potent free radical scavenger and showed the highest protection factor against DPPH^•^, ABTS^•+^, H_2_O_2_ and superoxide free radicals. Its activity is comparable to the synthetic antioxidant BHT and Trolox clearly superior to natural ascorbic acid and α-tocopherol (Figures 2–5).

Natural compounds are receiving increasing attention as potential antioxidants. For this purpose, luteolin 7-glucoside was the most dominant compound followed by hesperidin. These compounds demonstrated scavenging properties toward the DPPH^•^, ABTS^•+^, H_2_O_2_ and superoxide free radicals, which indicated their ability to efficiently scavenge free radicals (Akdemir et al. 2003, 2004a, 2004b).

The high values of LD_50_ of 8200 and 4225 mg/kg b.w. of MeOH and *n*-BuOH extracts, respectively, indicated their safety. In the acute toxicity in male rats of the MeOH and *n*-BuOH extracts of *V. nubicum* orally at dose 12,000 and 7000 mg/kg b.w., respectively, there were no changes in animal behavior, but the body weight gains were significantly different in the treated rats. Since, the changes in animal behavior have been used as an indicator of adverse effects of drugs and chemicals (El Hilaly et al. 2004). The present results suggest that at the oral dose, the MeOH and *n*-BuOH extracts are safe.

Indomethacin has been found to interfere with the synthesis of proteoglycans by chondrocytes, trans-membrane ion fluxes and cell to cell binding. It also has the ability to unmask T-cell suppressor activity that may lead to a reduction in the rheumatoid factor (Nwafor et al. 2000). Several plants containing high amounts of saponins have been shown to possess anti-ulcerogenic activity in several experimental ulcer models. The protective activities of these saponins may be due to the activation of mucous membrane protective factors and inhibition of gastric secretion volume and acid secretion. Many phytochemical analyses led to the isolation of mucilage, flavonoids, phenylethanoids and saponins from the inflorescence of some *Verbascum* species. Consequently, data on the phytochemistry of *Verbascum* species suggested that investigation of the anti-ulcerogenic activity against ethanol-induced gastric ulcer model in rats. However, no rat was completely protected from any visible damage (Gurbuz et al. 2005). In this study, the MeOH and *n*-BuOH extracts of *V. nubicum* were shown to protect rats from developing gastric ulcer induced by indomethacin in a dose-dependently manner.

It has been proposed that mucosal protection induced by non-prostaniod compounds may be mediated through the mobilization of endogenous prostaglandins (Cho et al. 1983; Konturek et al. 1998; Nwafor et al. 2000). Prostaglandins are a potent mediator of inflammation that results in edema, pain, and vasodilation; it also influences other mediators of inflammation including the synthesis of leukotrienes, superoxide generation and lysozomal enzyme release. Prostaglandin E2 protects the gastric mucosa from acid damage by maintaining adequate blood flow (Caceres et al. 1991), Presence of flavonoids such as luteolin 7-glucoside, hesperidin and apigenin have been reported to offer some protection in ulcer development by increasing capillary resistance and improve microcirculation which renders the cells less injurious to precipitating factors (Caceres et al. 1991).

The MeOH and *n*-BuOH extracts of *V. nubicum* were shown to possess significant inhibitory activity in the formalin-induced hind paw edema model and in acetic acid-induced writhings in rats. Through a bioassay-guided by HPLC analysis which indicated the presence of luteolin 7-glucoside, hesperidin and apigenin. All these compounds were found to possess significant antinociceptive and anti-inflammatory activities, per- or without inducing any apparent acute toxicity or gastric damage. Results of these study supported the continuous utilization of this species employed in folk medicine (Kupeli et al. 2007). The present study was in agreement with the results of Hartleb and Seifert (1995). The anti-inflammatory effect of *V. nubicum* extracts observed in our experiments may be related to the presence of its flavonoid content. The presence of polyphenols and flavonoids as major components could be the reason for the anti-inflammatory activity as explained by Bai et al. (2013). Thus, these compounds may contribute to the antinociceptive and anti-inflammatory activities reported here and the combination of these two properties could help to support the usefulness of the plant in the treatment of arthritis and thus validate the traditional uses of this plant.

The hepatoprotective activity of *V. nubicum* was tested using LPS-induced liver injury, caused a significant increase in the hepatic ALT, AST, LDH, TBARS, (TNF-α) and NO compared to the control, Tables 6 and 7. Our results were in agreement with Abd-Allah (2006), who reported that the administration of LPS caused a significant increase in ALT, AST, LDH, TBARS, (TNF-α) and NO. However, there was a decrease in the activity of hepatic GPx, SOD and CAT as well as GSH levels when compared with control rats. Elevation of hepatic NO level by LPS administration in experimental rats could be the reason for the activation of iNOS expression in the Kupffer cells, which could impair hepatic function (Kaur et al. 2006).

Phytochemical studies in the present study have shown that the unique culinary and medicinal properties of *V. nubicum*, may be attributed to its polyphenols and flavonoids content. Our biochemical results in agreement with many studies which proved the hepatoprotecive activity of these polyphenolic compounds.

Many studies reported that hepatoprotecive effect of luteolin against various kind of insults like d-galactosamine, CCl_4_ and *N*-nitrosodiethylamine in animal models (Domitrović et al. 2009; Lee et al. 2011; Balamurugan and Swamidoss 2010). Also, hesperidin is a pharmacologically active bioflavonoid found in many of the medicinal plants, with antioxidant and free radical scavenging property (Pari et al. 2015). In our present study MeOH and *n*-BuOH extracts of *V. nubicum* treatment ameliorate the LPS associated rise in TBARS, ALT, AST, LDH and depletion of antioxidant enzymes (CAT, SOD and GPx) as well as GSH level. This could be attributed to the scavenging activity of MeOH and *n*-BuOH containing hesperidin towards ROS and sparing of endogenous antioxidant enzymes (Pari et al. 2015). Hydrophilic glycosidic part of hesperidin helps it to reside in the cytoplasmic compartment of cell. So this bioflavonoid has a good intracellular radical scavenging activity and is able to inactivate reactive metabolites and ROS at the site of its production itself (Hwang et al. 2012). Thus it can prevent the peroxidation of polyunsaturated lipids present in the plasma membrane and other subcellular organelles like endoplasmic reticulum, mitochondria and helps to maintain its integrity (Nathiya et al. 2015).

Our HPLC analysis of MeOH extract revealed the presence of certain bioactive components, it was obvious from the results that the butanol extract effect is more pronounced than MeOH, it can, therefore, be said that these bioactive constituents of the plant reside mainly in the butanol extract more than MeOH (Armatu et al. 2011).

Histological examination of liver sections of MeOH and *n*-BuOH extracts of *V. nubicum* groups revealed a decrease in hepatocytes necrosis and inflammation than that of LPS-treated group. The *n*-BuOH group showed a more pronounced effect than MeOH group. This effect may be due to the reason mentioned earlier (Armatu et al. 2011). Both extracts preserved the structural integrity of hepatocellular membrane, with no signs of hemorrhage or apoptosis.

In addition, the data in Table 8, showed an increase in the peritoneal macrophages count/mL, as well as significant elevation of TNF-α, NO and PGE_2_ and COX-2 in the peritoneal macrophages of the LPS-treated rats compared to normal control. However, inflammatory mediators generated by the endothelial cells is mostly considered responsible for inflammation-induced injury, the migration of the inflammatory cells might be the potential source of TNF-α, NO and PGE_2_ and COX-2 inflammatory mediators at the site of inflammation (Sethi and Dikshit 2000). The previous study stated that the concentration of TNF-α, NO in the circulation was proposed as a reliable marker for the morbidity and mortality of the patients, as well as experimental animals (Hwang et al. 2007). Our results were in agreement with (Abd-Allah 2006; Dong et al. 2015; Wahby et al. 2015) who reported that LPS caused a highly significant increase in the hepatic inflammatory mediators in the peritoneal macrophages of the LPS-treated rats compared to control.

## Conclusions

To our knowledge, this study is the first report for the isolation of gentiopicroside, luteolin, aucubin and gallic acid, respectively, from *Verbascum nubicum*. HPLC analysis of methanol extract was accomplished to investigate the flavonoids and phenolic profile of this plant. Also, this study is the first report on the characterization of *V. nubicum* biological activities. The present study showed that *V. nubicum* MeOH and BuOH extracts significantly inhibited the production of pro-inflammatory mediators, including TNF-α, NO and PGE_2_, COX-2 enzyme activity in LPS-induced inflammation in rats and peritoneal macrophage cells. Moreover, our results proved that the hepatoprotective activity and the antioxidant potential effect of the plant. Taken together, these results suggest that *V. nubicum* may be a new commercial product in the drug industry.
